# Clinical microbiology laboratories and COVID-19: an interview with Joseph Blondeau

**DOI:** 10.2217/fmb-2021-0113

**Published:** 2021-06-04

**Authors:** Joseph Blondeau

**Affiliations:** ^1^Division of Clinical Microbiology, Royal University Hospital & Saskatchewan Health Authority, Saskatoon, SK, Canada; ^2^Department of Microbiology & Immunology, Pathology & Laboratory Medicine, Ophthalmology, University of Saskatchewan, Saskatoon, SK, Canada

**Keywords:** antimicrobial research, clinical microbiology, clinical microbiology laboratories, COVID-19, COVID-19 testing, diagnostic laboratories, SARS-CoV-2

## Abstract

This interview was conducted by Atiya Henry, Commissioning Editor of *Future Microbiology*.

Joseph M Blondeau, MSc, PhD, RSM(CCM), SM(AAM), SM(ASCP), FCCP is a Clinical Microbiologist and Head of Clinical Microbiology at Royal University Hospital (Saskatoon Health Region) and the University of Saskatchewan in Saskatoon, Canada. He is also the Provincial Clinical Lead for Microbiology in Saskatchewan, Canada. He holds a Masters of Sciences in Microbiology from Dalhousie University (1985) and a Doctor of Philosophy in Medical Microbiology from the University of Manitoba (1989). Following completion of his PhD, he completed a 1 and a half year post-doctoral training in an infectious diseases research laboratory at Dalhousie University and following which he completed a 2 year post-doctoral residency training program in Clinical Microbiology, also at Dalhousie University. He holds appointments as a Clinical Associate Professor of Pathology, Adjunct Professor of Microbiology and Immunology and Clinical Associate Professor of Ophthalmology. He teaches to undergraduate and graduate students in the areas of microbiology, infectious diseases, antimicrobial agents and pharmacology. Dr Blondeau’s main research interests are in the area of antimicrobial agents and antimicrobial resistance, clinical microbiology and clinical outcomes associated with antimicrobial therapy in both human and veterinary medicine.

## For those readers unfamiliar with yourself, could you please begin by introducing yourself, your background & tell me a bit about your work to date?

I am a clinical microbiologist and I work in a diagnostic clinical microbiology laboratory. I am head of the Clinical Microbiology Service at Royal University Hospital and the University of Saskatchewan in Saskatoon, Canada. I am also the provincial lead for Clinical Microbiology with the Saskatchewan Health Authority for the province of Saskatchewan. My career is one that has evolved to not only being a laboratory diagnostician in regards to clinical microbiology, but also an externally funded research program focusing on antimicrobial and diagnostic technology research. Finding better ways to diagnose infections in the lab more efficiently improves patient care and as well the global concerns with antimicrobial resistance and multidrug-resistant pathogens requires continuous research. Since our research program was started we have raised 4.5–5 million dollars in research money – primarily for antimicrobial research. St. Paul’s and Royal University Hospital in Saskatoon are the only places I have ever practiced and I was recruited here while I was in training; I trained at Dalhousie University in Halifax, Nova Scotia and at the University of Manitoba in Manitoba, Canada. I was recruited to Saskatoon after I did my clinical microbiology fellowship training and have been here ever since; my 30th anniversary in April 2021.

## What would you say has been the most memorable & exciting moment of your career?

It is hard to pick one, but I do recall a number of years back we had a woman who was on the kidney transplant list for 12 years, and an eligible donor became available. One of my laboratory technologists and I worked throughout the night in order to make sure all the testing was done so that she could receive her kidney the next day, and it was successful. I think that is probably one of my greatest moments of satisfaction, recognizing that we were finally able to help somebody who had been waiting for so long. We recognized, we were only a small part of that success story but we had to do our part in order for this to move forward. Of course, now the global COVID-19 pandemic has impacted our diagnostic services in so many ways. The resilience and adaptability of our staff has been nothing short of amazing.

In terms of my research career, there has been many highlights. One of the things I found was, sometimes when we get presented with problems we have to think outside of the box and decide how to design an experiment or protocol that would allow us to address a question. Saskatoon is a city of bridges that cross over the river in many places and sometimes when I had a problem, I would go out running. I remember once running across one of the bridges and it came to me how we could resolve a problem, and so I rushed back and wrote everything down. We have since published that solution, so sometimes when you clear your head and then try and address a problem, things come to you. I have had a number of moments like that in my career and corresponding research publications.

## Last year, you wrote a *Future Microbiology* article entitled ‘Clinical microbiology laboratories & COVID-19: the calm before the storm’ where you spoke about the challenges faced by diagnostic laboratories. What inspired you to write this piece?

The inspiration for that piece [1] was based on my day-to-day life in a diagnostic lab, struggling to deal with the COVID-19 pandemic and demands for testing. Our model here is we huddle every morning with all the members of the team, such as laboratory technologists, clinical staff etc. and we look at our challenges for the day – do we have full staffing, are there any problems at any of our work benches that require additional support etc. and specimen numbers. Historically, our labs were staffed for the demands that we regularly face prior to COVID-19. We normally processed 1000–1400 patient specimens a day, so we quickly recognized based on modeling projections, we could see the number of daily specimens double or even triple. We had challenges with regarding how we get staff to handle this additional volume and updating our existing technology and buying new technology to allow us initially to handle this increased volume. We were already operating 24/7, but we were not doing COVID testing 24 h a day.

Also, we had appropriate clinical pressure from the intensive care unit and from the emergency department. They had patients that were waiting for COVID-19 results. If the specimen came in at 10 pm, they were not going to get a result until sometime the next day and that just was not satisfactory. All of these demands were daily struggles for our team, and how we addressed them was the inspiration for that article as were the solutions. I felt others might benefit from some of our experiences when I wrote it.

## In your article, you mentioned that the main challenges were staffing, technology, training & supply chains. In the past 6 months, what new challenges have you faced & how have you had to adapt?

We have faced new challenges, but not just because of COVID-19. If you review the peer reviewed literature [2,3], you will recognize that patients with COVID-19 who are admitted to the hospital may have other diagnostic tests such as blood cultures, sputum cultures etc. ordered. Or, they could have respiratory specimens collected to be analyzed for other viruses. So, one of our struggles was that, the COVID test was a single test and it was not blended in with other assays we do for other respiratory viruses. So if it is not COVID and clinicians wanted to know if it is possibly influenza or another respiratory virus, we would retest using another assay, thereby increasing workload. So if I was to say we have 1000 extra specimens today, those 1000 extra specimens might be not just for testing COVID but for COVID and something else; thereby, compounding the testing. Some of the new technology that we have purchased has not all yet been validated and implemented because we had to get all the training and implementation done on the equipment that was most critical. We also recognized that there was other technology that we could afford to delay validation, training and implementation.

The dynamics of the pandemic are ever present and along comes the variants. Once again more adjustments ensure we are correctly detecting the variants. We have expanded our in-house molecular assays in order to provide more real time results. Again, retooling and ‘on the fly’ adjustments to accommodate these new challenges.

## What advice would you give to other clinical microbiology labs on how to manage the strain of the increase in specimen numbers?

You really only have two choices here, you have to build business cases in order to request additional resources and those resources will be people or technology, or both. You need to build into your business case the amount of time that is required to validate and train individuals on an instrument and then an operating costs going forward. Any advice I would give to other labs would be ‘do not be afraid to ask’. In other words, if your administrators or if your government or whatever the case may be, wants a certain level of testing to be done, they have to recognize that there is a cost to providing such services, and then ask for the resources. In our province, and as the Provincial Lead for Microbiology, I was constantly working to get technology for our own institution along with the addition of technology to other sites around the province providing microbiology services. We were very successful in getting substantial amounts of funding to get the necessary equipment – so do not be afraid to ask is my advice. If they come back and say you cannot have it then you have to say: ‘what will we not do, in order to do what it is that you want?’ You cannot do everything if you are not resourced to do it – it's that simple.

## Laboratories that offer 24/7 microbiology services are vital for timely diagnostic results: what do you think are the key obstacles that prevent labs from operating around the clock? How can we overcome those obstacles?

We attempted to address this. In an earlier Editorial that I copublished with Evgeny Idelevich, we talked about 24 h clinical microbiology service being essential for patient care [4]. One of the biggest barriers is administrative support. Sadly, we did have opinions questioning the value of microbiology results in the middle of the night but it is clear that a critical result (i.e., blood culture) could immediately impact patient care. If a hospital administrator says they do not have the funding to staff the laboratory 24 h a day, there is little you can do except continue to make the argument regarding quality patient care and the potential savings (i.e., other unnecessary diagnostic tests) from a more timely diagnosis. There are very strong arguments a person could make to show the benefits of having 24-h service. For instance, we report positive COVID results between midnight and 7 am. If we did not have somebody working here throughout the night, those results would have to wait until the morning – this has an important impact on hospital beds, patient flow and potentially discharges. We also provide services to intensive care, emergency and inpatient services in the middle of the night, in other words, reporting positive blood cultures within an hour of detecting them positive in the lab. I think it is an easy argument to make that labs providing 24/7 service are optimizing the laboratory support for patient care, but administrative support and the finances necessary are probably the biggest obstacles.

## You previously briefly touched on the phenomenon of ‘COVID fatigue’. What steps do you think can be taken, to reduce the risk of burnout in laboratory technologists & other frontline staff?

We believe here that it is essential for us to be operating with a full slate of staff, because the division of labor is much better than if we are operating with a reduced number of staff. ‘Burnout’ is a legitimate concern. Our medical laboratory technologists have to go through a training program to make them proficient on the different benches, for example, the blood culture bench is different than the wounds bench, closed-space bench or the respiratory bench etc. One of the ways we are preventing burnout is by not getting people to do the same thing every single day – to provide them with a variety of things they can be doing, so that they are getting that satisfaction from their job by using all of their skills. As well, we have made temporary but substantial increases to the number of staff doing testing. We have had to hire staff with different skill sets and then train them to perform the testing required. This was essential to meet testing demands.

## With the rollout of COVID-19 vaccines in progress, how do you think this will impact the workload at your laboratory?

I think over time we will probably see that workload go down, unless the variants that have evolved and if they continue to evolve, get to a point where we have to continue testing for variants versus just regular old COVID-19. I expect with vaccination over time, the demand for COVID testing is going to go down. That is my prediction, whether I am right or wrong who knows. What is going to continue to influence this is, whether or not variant strains continue to arise and whether we have a need to test for those, particularly if some of those are less well covered by the vaccines. If a new variant or some of the existing variants are proven to need a new vaccine, I think testing will continue for the foreseeable future. On the flip side, if we get to a point where variants predominate, testing specifically for variants might be unnecessary and widespread population testing will decline.

The other thing we do not know is whether COVID-19 will become a seasonal virus the same as other coronaviruses, and if that is the case then we would anticipate that we would combine COVID testing into a multiplex assay where we would test it alongside influenza and other viruses. This has, in fact, already happened and we test COVID and influenza simultaneously in one assay. We also have a 16-plex assay which detects 16 different viral targets – COVID is not part of that, but we could anticipate a similar type of assay in the future.

COVID-19 has been nothing short of extraordinary and diagnostic clinical microbiology laboratories have been front and center to this pandemic.
